# Notes and News

**Published:** 1896-06-06

**Authors:** 


### Notes and News.

(Collected and Communicated.)

### HOSPITAL MEETINGS.

The Annual General Meeting of the Alexandra Hospital for Children with Hip Disease will be held at the Hospital on Monday, June 8th, at 4.30.

A Congress of Hygiene and Medicine will be held at Budapest in September, which will be devoted to the study of national needs in these directions.

A Boston merchant has given 100,000 dols. to endow a chair of Comparative Pathology in Harvard University.

The foundation stone of a hospital for the town of Aldershot will be laid, in July, by the Duchess of Connaught, who has also consented to receive purses with donations for the building fund.

At the annual general meeting of the Royal Medical Benevolent College, Sir Arthur Watson, Bart., Q.C., and the Right Hon. A. J. Balfour, M.P., were elected vice-presidents of the institution.

Sir William Broadbent will preside at the festival dinner on June 17th in aid of the Clarence Memorial Wing to be added to St. Mary's Hospital. Sir William Broadbent is about to retire from his post of senior physician to the hospital.

A bust has been placed to the memory of the late Professor Andrea Vega, in the Institution of the Society for the Protection of the Insane Poor, at Milan. This society, like most of its kind, was the outcome of the energy of the great Italian alienist.

Dr. Leith Napier has been appointed senior surgeon, gynecologist, and lecturer on clinical surgery and gynecology at the Adelaide General Hospital, for whence he sails immediately. Dr. Napier's new and important work on "The Menopaure and its Disorders" will shortly be issued by the Scientific Press.

Such victims of the terrible disaster at the coronation festivities as were removed to the hospitals in Moscow were visited by the Tsar.

A competitive examination for twenty-five commissions in the Army Medical Staff will be held on August 7th next and following day. Particulars thereof will be found in our advertisement columns.

Mr. Cory-Wright, the deputy chairman of the Great Northern Central Hospital, has endowed a bed at that institution, and has promised a further £150 towards the endowment of another bed if eighteen donors of £50 each come forward before June 11th.

A new wing at the Convalescent Home at West Kirby has been opened recently. It contains isolation wards, and one for crippled children, also a disinfecting chamber and day-room. Thirty additional beds are provided, the cost of the building being £3,000.

The council of the Metropolitan Hospital Sunday Fund wish it to be known that they do not sanction street collections on behalf of the fund. Those who do not attend divine service on Hospital Sunday are asked to send their contribution to the Mansion House direct.

The Jenner Society, the headquarters of which will be at Gloucester, is now established. The Earl of Ducie is the president, and many distinguished local personages form the Council. The main objects of the society are to protect the memory of Dr. Edward Jenner, and to put vaccination on a more satisfactory basis than it at present rests.

Two memorial stones have been laid in connection with the intended hospital for Kettering. One stone has been laid by the Duchess of Buccleuch and the second by Lady Brunner. The site for the hospital has been given by the Duke of Buccleuch, and the estimated cost of the building is £10,000. So far £6,300 has been raised.

A new wing has been added to the Heatherdene Convalescent Home connected with the Sunderland Infirmary. The Home was opened in 1892 for the reception of women and children, but the extension will now permit of the reception of male patients. The new wing has cost about £4,500. At the opening ceremony Mr. Laing and Mr. Alderman Richardson, both hearty friends and benefactors of the institution, were presented with portraits. Mr. Laing received a portrait of his late son, and Alderman Richardson one of himself.

The comparative absence of small-pox from the metropolis at a time when the terrible epidemic at Gloucester has engendered renewed terror at the scourge is very satisfactory. Nor does Gloucester itself appear to have disseminated the disease elsewhere, though anyone coming from that infected city is regarded with very grave suspicion, and exaggerated rumours have been spread from time to time. In one small town in Hampshire where a fair was being held it was reported that two holiday-makers had fallen off a roundabout victims of infection brought by a travelling showman, who, it appeared, had visited Gloucester some months previously.

